# Reduction of endoplasmic reticulum- mitochondria interactions in beta cells from patients with type 2 diabetes

**DOI:** 10.1371/journal.pone.0182027

**Published:** 2017-07-25

**Authors:** Charles Thivolet, Guillaume Vial, Romeo Cassel, Jennifer Rieusset, Anne-Marie Madec

**Affiliations:** 1 INSERM UMR-1060, CarMeN Laboratory, Lyon 1 University, INRA U1235, Lyon, France; 2 Hospices Civils de Lyon, Lyon-Sud Hospital, Department of Endocrinology and Diabetes, Pierre-Bénite, France; Children's Hospital Boston, UNITED STATES

## Abstract

Type 2 diabetes develops when beta cells are not able to fulfill insulin needs. The role of the endoplasmic reticulum–mitochondria junction in coordinating the functions of these two organelles throughout the natural history of type 2 diabetes is determinant and may explain the alterations of insulin biosynthesis. Our goal was to study endoplasmic reticulum and mitochondrial interactions in human beta cells from organ donors with type 2 diabetes. Pancreas samples were obtained via the network for pancreatic organ donors with diabetes (nPOD) based on disease status with 12 subjects with type 2 diabetes and 9 non-diabetic controls. We examined pancreatic specimens by immunofluorescence, *in situ* hybridization and *in situ* proximity ligation assay and compared the results to an *in vitro* model of beta-cell dysfunction. Expression of proteins that enable tethering and exchanges between endoplasmic reticulum (ER) and mitochondria and quantification of interconnection through mitochondria associated membranes (MAM) was investigated. In beta cells from type 2 diabetic cases as compared to controls, there was a significant increase in reticular expression of inositol triphosphate receptor-2 (IP3R2) both at the protein and mRNA levels, no difference in mitochondrial transit peptide receptor TOM20 and mitofusin-2 expressions, and a decrease in the expression of voltage-dependent anion channel-1 (VDAC-1). The number of IP3R2-VDAC-1 complexes identified by *in situ* proximity ligation assay was significantly lower in diabetic islets and in beta cells of diabetics as compared to controls. Treatment of Min6-B1 cells with palmitate altered glucose-stimulated insulin secretion, increased ER stress and significantly reduced ER-mitochondrial interactions. We can conclude that specific changes in reticular and mitochondrial beta cell proteins characterize human type 2 diabetes with reduction in organelle interactions. This finding opens new targets of intervention.

## Introduction

Type 2 diabetes is characterized by beta cells being unable to produce sufficient amounts of insulin in the context of insulin resistance. Decreased beta cell function and mass predict diabetes onset and progression [1]. A better understanding of beta cell function during type 2 diabetes is essential to develop early interventions and to prevent the consequences of chronic exposure of beta cells to high glucose. During the last 30 years, important efforts have been made to understand the contribution of beta cells to the pathogenesis of type 2 diabetes but this remains a matter of debate due to limited access to the pancreas in humans and difficulties in imaging beta cells *in vivo* and to correlate dynamic testing with beta cell mass. Interrelated stressors alter beta cells with different proportions among diabetic individuals that include lipid accumulation, inflammation, endoplasmic reticulum (ER) stress, oxidative stress and amyloid deposits which ultimately lead to beta cell apoptosis [1, 2]. This sequence of events are largely extrapolated from animal models as well as from *in vitro* studies using cell lines or primary islet cell cultures and reinforces the need to intervene as early as possible in the course of the disease to prevent beta cell loss. A reduction in beta cell mass has been found in several studies of autopsied pancreata [2, 3] but accurate levels are difficult to compare due to differences in pancreatic parenchyma volume in obese vs lean subjects [4] and according to duration of disease. There is also an important heterogeneity among patients due to genetic polymorphisms that control insulin synthesis as well as disparities between the levels of nutritional load and body’s insulin sensitivity.

In the context of insulin resistance and type 2 diabetes, it is admitted that beta cells are unable to increase insulin output to maintain glucose tolerance and that progressive functional changes precede cell apoptosis [5]. ER and mitochondria are two metabolic organelles playing a key role in beta cell function. Activation of ER plays a crucial role in the synthesis, correct folding and sorting of insulin in response to glucose. ER forms the main intracellular Ca2+ reservoir and the controlled release of Ca2+ into the cytosol is a critical step for insulin synthesis. Intracellular compartments have to exchange material and transmit signals between each other to maintain and balance cellular activities. A special ER compartment communicates with mitochondria and functionally interacts at sites defined as mitochondria-associated membranes (MAM) in order to fulfill a plethora of functions associated with, among others, lipid metabolism and Ca2+ signaling but also the regulation of mitochondrial maintenance and programmed cell death/cell survival [6]. Among the group of proteins enriched at ER-mitochondrial interface, inositol 1,4,5-trisphosphate sensitive Ca2+ channels (IP3R) regulates calcium release activities through an interaction with the GRP75/VDAC-1 complex [7]. Increased basal and decreased glucose-stimulated Ca^2+^ concentrations have been associated with cell dysfunction [8] and alteration of beta cell calcium dynamics is an early event during type 2 diabetes [9]. Whereas recent data suggested a disruption of organelle coupling in insulin resistant liver that may participate in altered glucose homeostasis [10,11], the relevance of MAM in beta cell dysfunction has not been studied.

The primary objective of the present study was to examine the levels of ER and mitochondria proteins that enable tethering and exchange of metabolites and signaling molecules. To address this, we analyzed at the mRNA and protein levels a series of high-quality human pancreatic tissues obtained from brain-dead organ donors with or without diabetes. We provided novel insights into beta cell dysfunction during human type 2 diabetes illustrating the importance of ER-mitochondrial interactions.

## Materials and methods

### Pancreatic sections

Formalin-fixed paraffin-embedded tissue sections from the tail of the pancreas from cadaver brain-dead organ donors were obtained through the Juvenile Diabetes Research Foundation (JDRF)-sponsored Network for Pancreatic Organ Donors with Diabetes (nPOD) program (http://www.jdrfnpod.org/for-investigators/online-pathology-information/). All procedures were in accordance with federal guidelines for organ donation and the University of Florida institutional review board. The nPOD collection of biological specimens for research purposes by noninvasive means was approved by the IRB from the University of Florida with the ref# IRB201600029 on 3/17/2016. Pancreas specimens were selected from donors with type 2 diabetes (n = 12) and donors with no history of diabetes (n = 9). Characteristics regarding the human tissue donors are presented in Table 1. The median age [range] from individuals with type 2 diabetes and controls were similar (45.3 [19–62] years vs 37.9 [14–65] years, t-test p = 0.46). The mean± standard error of the mean (SEM) BMI was significantly higher in individuals with type 2 diabetes (34.58±1.46 vs 26.21±1.74, Mann Whitney test p<0.01). The mean pancreatic weight obtained from nPOD registry was similar among the two groups of donors. It is interesting to note that mean±SEM peripheral C-peptide values were significantly lower in patients with type 2 diabetes (4.17±1.83 vs 8.34±1.49 ng/ml, p = 0.02).

**Table 1:** Characteristics of the 12 pancreatic donors with type 2 diabetes and the 9 pancreatic donors with no history of diabetes (controls).

Type 2 diabetes	Age	BMI	Sex	Race	Duration of Diabetes (years)	C pept (ng/ml)	Pancreas weight (g)	Insulin therapy
**6028**	33	30.2	M	AFR AM	17	22.4	49	yes
**6059**	19	39.1	F	HISP	0.25	10.68	74	yes
**6108**	58	30.4	M	ASIAN	2	1.25	91	no
**6110**	21	40	F	AFRIC	0.5	0.58	69	
**6114**	43	31	M	CAUC	2	0.58	100	no
**6124**	62	33.7	M	CAUC	3	2.85	104	no
**6127**	45	30.4	F	CAUC	10	0.08	59	yes
**6132**	56	44.6	F	HISP	-	0.8	70	
**6133**	46	40.2	F	CAUC	20	0.84	70	yes
**6221**	61	33.7	F	CAUC	4	3.05	74	no
**6249**	45	32.3	F	ASIAN	15	4.17	54	yes
**6255**	55	29.4	M	CAUC	6	2.78	104	no
**Controls**								
**6009**	45	30.6	M	CAUC		11.32	-	
**6013**	65	24.2	M	CAUC		2.8	34	
**6057**	22	26	M	CAUC		16.23	104	
**6179**	21	20.7	F	CAUC		2.74	72	
**6229**	31	26.9	F	CAUC		6.23	46	
**6233**	14	21.9	M	CAUC		7.26	61	
**6235**	30	25.4	M	CAUC		8.1	102	
**6288**	55	37.7	M	CAUC		12.96	111	
**6290**	58	22.5	M	CAUC		7.46	86	

### Cell culture

Min6-B1 cells were cultured in DMEM (20mM/l glucose) supplemented with 15% of heat-inactivated fetal bovine serum, 2 mM glutamine, 100 U/ml penicillin, 100 μg/ml streptomycin and 71 μM beta-mercaptoethanol (Sigma Aldrich, St-Quentin Fallavier, France). For lipotoxicity studies, Min6 cells were treated with bovine serum albumin (BSA) or palmitate (conjugated to BSA at a 6:1 fatty acid to BSA molar ratio, 200μmol/L for 24 h) in serum-free culture medium. At the end of the 24 h incubation period, Min6B1 cells were kept for 1h in Krebs-Ringer bicarbonate (125 mM NaCl; 4.7 mM KCl; 1 mM CaCl2; 1.2 mM MgSO4; 1.2 mM K2HPO4; 5 mM NaHCO3; and 25 mM HEPES 132 pH 7.4) supplemented with 0.5% bovine serum albumin and 2.7 mM/l glucose then challenged with 16.7 mM/l glucose to assess glucose-stimulated insulin secretion (GSIS). Insulin was measured by a specific immuno-radiometric assay (Bi-insulin IRMA, Cis-Bio International, Gif sur Yvette, France). Genomic DNA was isolated from MIN6B1 cells using the standard phenol/chloroform method. The relative mtDNA content was measured by quantitative real-time PCR (qPCR). The primer pair COX1 (5’- ACTATACTACTAACAGACCG-3’) and (5’- GGTTCTTTTTTTCCGGAGTA-3’) was used for mtDNA detection. The amplification of the primer pair PPiA (5’—ACACGCCATAATGGCACTGG-3’) and (5’-CAGTCTTGGCAGTGCAGAT-3’) was used for nuclear DNA normalization. The values were expressed as mtDNA/nDNA ratios.

### Immunofluorescence

Pancreatic slides were deparaffinized with Slidebrite (BioCare Medical, Concord CA) and dehydrated using graded ethanol concentrations. Slides were boiled in antigen retrieval citrate buffer pH6, blocked for 1hr with 2% normal goat serum, 2% bovine serum albumin, 0.5% Tween 20 in phosphate buffer saline followed by incubation with primary antibodies overnight at 4°C using different combinations. The staining series included antibodies to IP3R2 (Abcam, Cambridge, MA, USA 1:50), Translocase of outer mitochondrial membrane 20 (TOM20) (Santa Cruz biotechnologies, Dallas Texas, USA 1:50), VDAC-1 (Abcam, 1:100), mitofusin-2 (Abcam, 1:100), insulin (Dako, Carpinteria, CA, USA 1:150), glucagon (Abcam, 1:200). Secondary antibodies coupled to a fluorochrome (AF488, AF555, AF647, Life Technologies, Grand Island, NY, USA) were added for 30 min at RT according to the species of primary antibodies. Nuclei were stained with Hoechst 33342 (Sigma-Aldrich) and preparations were mounted in Prolong Gold anti-fade reagent (Life Technologies). Slides were analyzed with a slide scanner AxioScan.Z1 (Carl Zeiss SAS, Marly le Roi, France) at x40 magnification.

### *In situ* hybridization

Sections were dried for 1 hour at 60°C, pretreated and hybridized with an hs-ITPR2 probe (Homo sapiens inositol 145-trisphosphate receptor type 2 (ITPR2) mRNA) for 2 hours at 40°C. Probes were custom-designed and labeled for use with RNAscope 2.5HD (Advanced Cell Diagnostics, Newark, CA, USA). Some sections were stained with Hs-PPIB or dapB probes as positive and negative controls, respectively. Amplification steps were performed prior to the detection of signals with 3,3′-diaminobenzidine. Sections were counterstained and mounted with Permount (Fisher scientific, Waltham MA, USA).

### *In situ* proximity ligation assay

Duolink II *in situ* proximity ligation assay (PLA) (Olink Bioscience, Uppsala Sweden) enables detection, visualization, and quantification of protein interactions (<40 nm) as an individual dot by microscopy. Primary antibodies to assess ER-mitochondria interactions were against IP3R2 (1:100) and VDAC-1 (1:200) as previously described [7]. Digitized slides were analyzed at x20 magnification. When necessary, beta cells and alpha cells were identified using anti-insulin and anti-glucagon antibody staining on the same slide. Dots were quantified in each islet using the Zen program and Fiji-ImageJ software and expressed as percentage of dots per nucleus. Experiments were performed at least twice using 2 to 5 non-consecutive slides for each donor. For Min6-B1 cultures, ER-mitochondria-interactions were assessed using a fluorescent PLA assay, employing antibodies directed against VDAC-1 (Abcam, 1:100) and IP3R1 (Santa Cruz laboratories, Dallas TX, USA 1:500) as described previously [10]. Experiments with Min6-B1 cells were performed at least three times, with a minimum of five fields taken per condition.

### Morphometric analysis

Stained sections were scanned at x20 or x40 magnification to create digital slide images using a Zeiss Axioscan Z1 slide scanner. Using the same sections, randomized islets were also examined with a Leica confocal SP5X microscope at x63 magnification for colocalization studies. The frequency of insulin positive islet cells was calculated as the ratio of insulin positive cells divided by the total number of islet cells of the sectional area. Intensities of each specific staining in insulin positive areas were determined using multi-fluorescence with the same acquisition parameters. Fiji-ImageJ software was used to quantify fluorescence intensities within beta cells and number of dots during *in situ* hybridization and *in situ* PLA.

### Real time PCR

Total RNA of Min6-B1 cells was extracted with the TRI Reagent Solution (Sigma-Aldrich). The levels of target mRNAs were measured by RT, followed by real-time PCR using a Rotor-GeneTM 6000 (Corbett Research, Thermo Scientific, Waltham MA USA). A standard curve was systematically generated with six different amounts of purified target cDNA and each assay was performed in duplicate. We measured TATA-binding protein (TBP) mRNA as a reference gene so that the results are expressed as a ratio referred to the expression of TBP and normalized to the control group.

### Statistical analysis

A normality test was performed for all data using D’Agostino and Pearson omnibus normality test included in the GraphPad Prism software (La Jolla, CA, USA). When distribution was normal, analysis was performed with parametric statistics, usually a Student’s t test. However, if values did not pass the normality test nonparametric statistics were used, specifically the Mann Whitney test. Box and Whisker quartile plots were performed with a specific macro (Add-Ins.com LLC, Hockessin, DE) with Microsoft Excel where boxplots are built considering the first and third quartile (25th and 75th percentiles) to define whiskers, the center bar is the median and the upper and lower bars the 1.5 sigma points. Data were expressed as the mean ± SEM. Statistical significance was defined as a value of p<0.05.

## Results

### Beta cells from patients with type 2 diabetes have higher expression of IP3R2

**Fig 1:**
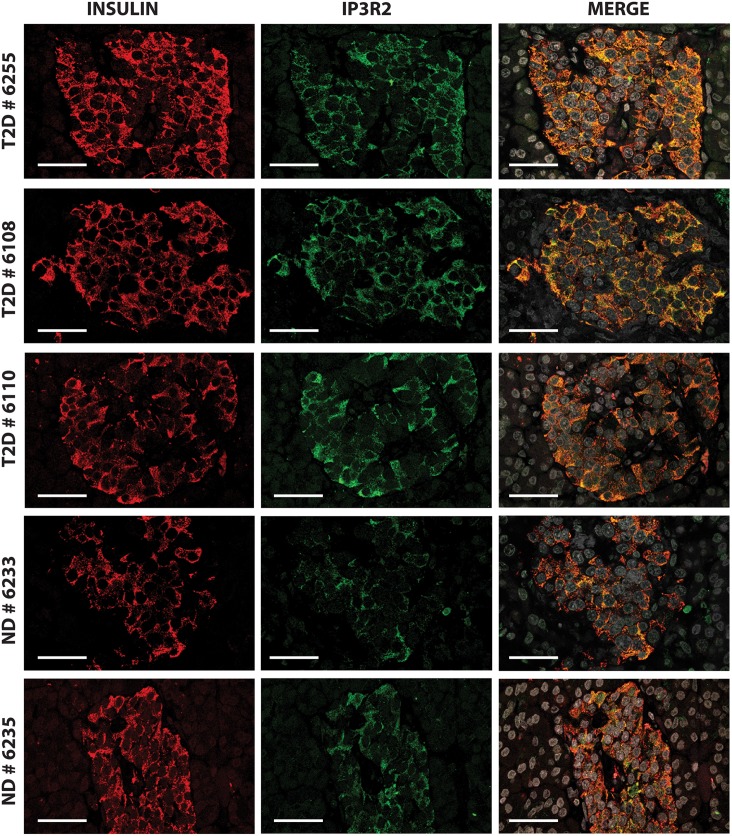
IP3R2 expression is enhanced in beta cells from diabetic donors. Confocal immunofluorescence images for insulin (red) IP3R2 (green), and merged stainings of pancreatic islets from three diabetic donors (nPOD# 6255, 6108, 6110) and two non-diabetic donors (controls; nPOD# 6233, 6235).

**Fig 2:**
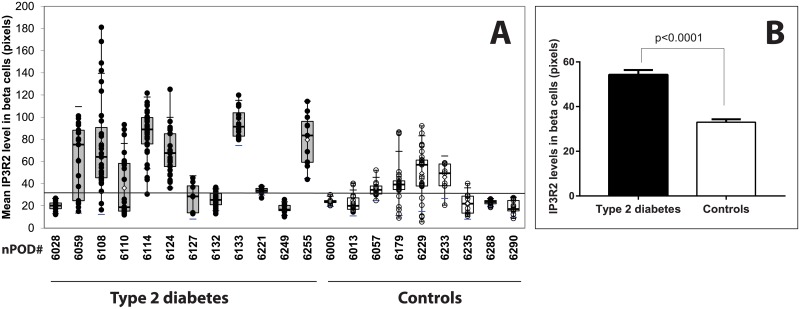
Morphometric analyses of IP3R2 expression in beta cells. (A) The expression of IP3R2 in beta cells was analyzed in 12 donors with type 2 diabetes (closed circles) compared to 9 donors without history of diabetes (open circles) by indirect immunofluorescence. Box and whiskers plots. Each circle corresponds to the mean expression level for a single islet. The horizontal line represents the upper 90^th^ percentile of control values. (B) Mean beta cell expression of IP3R2 levels were significantly higher in subjects with type 2 diabetes in comparison to controls (Mann Whitney test, p<0.0001).

Because beta cells are subjected to high insulin demand in response to hyperglycemia and peripheral insulin resistance, we postulated that expressions of proteins involved at the ER-mitochondrion interface were increased during type 2 diabetes. Since ER is the main intracellular Ca^2+^ reservoir, we first studied the expression of the IP3R, an important channel through which Ca^2+^ is released from the ER. We decided to study the expression of the IP3R2 isoform since we demonstrated that IP3R2 was colocalized with insulin (Pearson's R value: 0.58, Spearman's rank correlation value: 0.46) as shown in S1 Fig. Type 2 diabetes was associated with a significant increase in the expression of IP3R2 in beta cells; a representative confocal pattern is shown in Fig 1 and representative images for each donor are shown in S2 Fig. Although the distribution of IP3R2 varied among islets and donors (Fig 2A), 7/12 subjects with type 2 diabetes had beta cells with mean IP3R2 intensities above the 90^th^ percentile of control values including some islets with high expression levels as shown in Fig 2A. The mean level of IP3R2 staining in beta cells from 12 donors with type 2 diabetes was significantly higher than in the 9 controls (mean ± SEM: 54.27±2.11 pixels from 261 islets from donors with type 2 diabetes vs 33.03±1.28 pixels from 178 control islets) using Mann-Whitney test (two tailed p value < 0.0001) as shown in Fig 2B. There was no correlation between IP3R2 expression and known diabetes duration or circulating C-peptide levels. We studied by *in situ* hybridization (ISH) experiments, the number of ITPR2 transcripts per islet cell with a representative figure shown in S3A Fig in comparison to positive and negative controls using PPIB and DapB probes, as shown in S3B and S3C Fig respectively. Expression of ITPR2 mRNA was significantly increased in islets from pancreatic donors in comparison to controls (mean ± SEM: 0.72±0.03 dots per islet cell from 97 diabetic islets vs 0.36±0.02 dots per islet cell from 76 control islets) as shown in S3D Fig using Mann-Whitney test (two tailed p value < 0.0001).

Mitochondria play important roles in energy production, Ca^2+^ exchange and cell death processes. We first investigated the level of expression of TOM20, a transit peptide receptor from the outer mitochondrial membrane (OMM). TOM20 expression in beta cells was similar between 11 type 2 diabetic donors (mean±SEM intensity: 57.37±2.83 pixels, n = 189 islets) and 9 controls (65.15±3.98 pixels, n = 164 islets; Mann Whitney test, p = 0.79) as shown in S4B Fig, with a representative pattern shown in S4A Fig. It should be noticed that mean intensities of TOM20 and IP3R2 staining were inversely correlated in Type 2 diabetic patients (Pearson r = -0.605, R squared 0.366, p<0.05) but not in controls (Table 2). Mean expression levels of VDAC-1, a MAM component from the OMM, were significantly lower in beta cells of 11 diabetic patients (mean value±SEM of 109.6±4.58 pixels (n = 216 islets) than in 8 controls (123±6.53 pixels, n = 125 islets; Mann-Whitney test, two tailed p value < 0.05) as shown in S5 Fig; a representative pattern is shown in S6 Fig. However, levels of VDAC-1 beta cell expression were heterogeneous among donors, including 5 subjects out of 11 with mean intensities above the 90^th^ percentile of control values, among whom 4 had concomitantly high IP3R2 expression levels (S5 Fig and Table 2).

**Table 2:** Mean intensity levels in beta cells of IP3R2, TOM20, MFN-2 and VDAC-1 by indirect immunofluorescence and levels of ER-mitochondrial IP3R2-VDAC1 interactions quantified by *in situ* proximity ligation assay (PA). Levels in diabetic donors above the 90^th^ percentile of normal values are in bold characters.

nPOD #	IP3R2	TOM20	MFN-2	VDAC-1	PLA
**6028**	19.86±1.61	**118.48**±**8.04**	28.33±1.73	32.85±3.10	1.44±0.08
**6059**	**61.46**±**7.77**	15.29±1.67	22.79±1.73	42.43±3.79	1.14±0.13
**6108**	**75.94**±**7.49**	44.15±6.33	**54.12**±**6.04**	**161.96**±**8.06**	1.13±0.05
**6110**	**63.05**±**6.00**	**75.68**±**6.05**	**66.72**±**4.03**	43.13±2.87	1.41±0.09
**6114**	**99.05**±**2.57**	48.80±3.46	**55.13**±**4.35**	**205.78**±**6.04**	0.6±0.03
**6124**	**68.06**±**3.31**	29.80±3.27	36.57±1.64	**196.33**±**9.63**	0.88±0.05
**6127**	27.64±4.61	31.97±4.21	33.78±2.56	ND	0.38±0.06
**6132**	22.54±0.94	**120.04**±**11.18**	26.23±1.74	**191.88**±**7.44**	ND
**6133**	**94.79**±**3.92**	20.01±2.10	34.86±0.56	**140.23**±**10.25**	1.92±0.06
**6221**	33.78±0.72	ND	15.20±0.79	89.65±4.83	1.00±0.05
**6249**	17.52±1.05	66.21±8.6	ND	95.25±6.42	1.33±0.07
**6255**	**79.55**±**7.01**	49.47±5.99	37.28±5.56	110.60±5.03	1.51±0.08
**MEAN DT2**	**54.27±2.11**	**57.37 ± 2.83**	**39.50**±**1.64**	**109.6 ± 4.58**	**1.18**±**0.03**
**6009**	24.13±0.89	16.36+0.94	17.78±1.02	130.83±15.41	2.05±0.14
**6013**	22.64±1.83	93.08±9.47	17.35±2.59	98.77±12.22	2.03±0.07
**6057**	35.23±2.02	68.56±2.41	25.44±1.92	64.85±5.47	2.5±0.11
**6179**	40.30±3.64	101.84±8.82	39.86±6.79	80.37±8.11	ND
**6229**	49.06±4.15	77.19±9.06	43.16±2.48	242.88±1.43	2.80±0.19
**6233**	45.85±4.26	23.08±5.57	36.45±1.04	65.52±2.02	1.22±0.06
**6235**	53.67±4.44	19.07±1.32	48.59±3.48	94.87±8.26	1.72±0.08
**6288**	23.04±0.53	19.17±4.42	58.54±5.13	ND	1.57±0.11
**6290**	18.74±1.88	134.08±7.38	31.52±3.42	74.76±2.81	2.55±0.18
**MEAN CTL**	**33.2**±**1.45**	**65.15** ± **3.98**	**38.07**±**1.36**	**123**±**6.53**	**2.10**±**0.06**
Lower 90^th^ percentile	30.8	58.56	35.82	112.2	2.02
Upper 90^th^ percentile	35.61	71.73	40.32	133.8	2.19
p (DT2 vs CTL)	<0.0001	ns	ns	<0.05	<0.0001

No correlation was found between TOM20 and VDAC-1 levels, or with known disease duration. Since MFN-2 is enriched at contact sites between ER and mitochondria, we also analyzed the expression of this tethering protein. Mean±SEM intensities of MFN-2 in beta cells were similar between 11 donors with type 2 diabetes (39.50±1.64 pixels, n = 214 islets) and 9 controls (38.07±1.36 pixels, n = 198 islets; Mann Whitney test, p = 0.70); individual islet values are presented in S7 Fig and a representative pattern of MFN-2 staining is shown in S6 Fig. Interestingly, 3/11 diabetic donors had mean MFN-2 intensities above the 90^th^ percentile of control values together with high levels of IP3R2 expression (Table 2).

### Type 2 diabetes is associated with a reduction of ER-mitochondrial interactions

**Fig 3:**
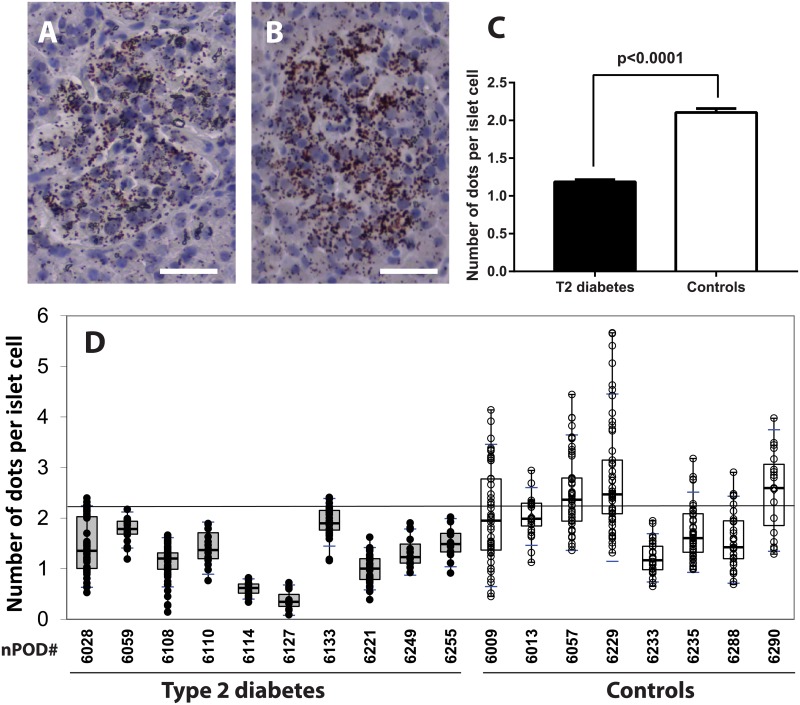
Quantification of IP3R2/ VDAC1 complexes in islets of diabetic donors and controls. (A) Representative bright field images of an *in situ* proximity ligation assay (PLA) are shown for a 33-year-old man who had type 2 diabetes for 17 years (nPOD# 6028) and (B) in a control (normoglycemic) 31-year-old woman (nPOD# 6229). (C) The mean number of dots per islet cell was significantly lower in donors with type 2 diabetes compared to controls. (D) Individual results from 11 donors with type 2 diabetes (closed circles) and 8 controls (open circles). The horizontal line represents the upper 90^th^ percentile of control values.

**Fig 4:**
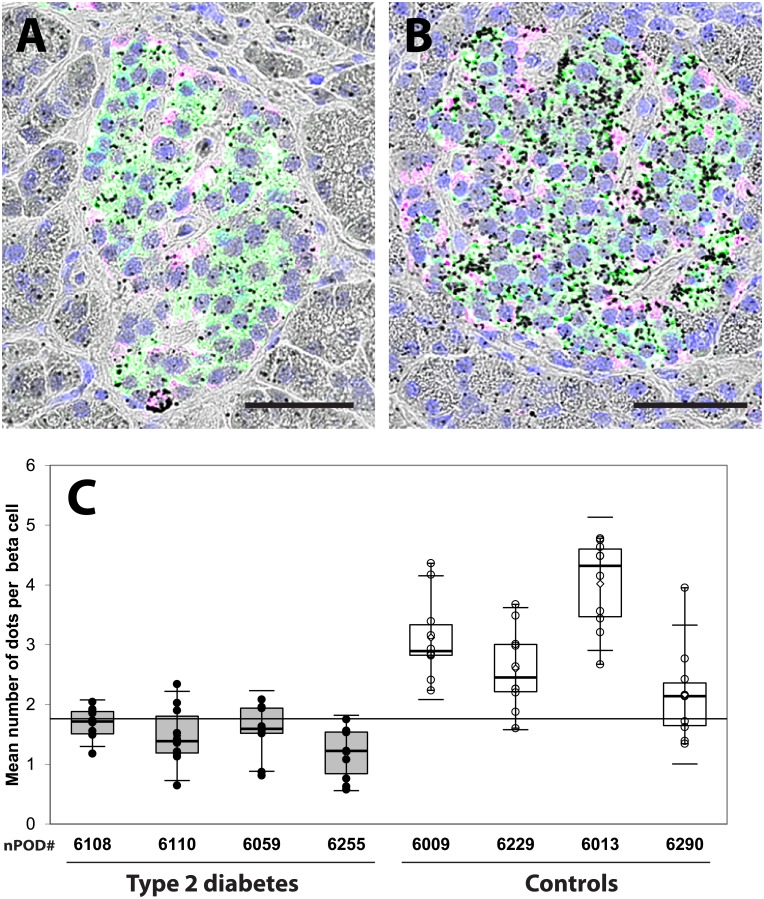
Quantification of IP3R2- VDAC1 complexes in beta cells of diabetic donors and controls. (A) Representative bright field images of an *in situ* proximity ligation assay (PLA) superimposed with insulin (green) and glucagon (purple) immunofluorescence stainings for a recently-diagnosed 19-year-old woman with type 2 diabetes (nPOD# 6059) and (B) in a control (normoglycemic) 31-year-old woman (nPOD# 6229). The measurement bar in A and B represents 50 microns. (C) The mean number of dots per beta cell was significantly lower in donors with type 2 diabetes compared to controls. The horizontal line represents the upper 90^th^ percentile of control values.

MAMs represent contact sites between ER and mitochondria and are important regulators of both organelle functions. To detect and quantify organelle interactions, we used an *in situ* PLA targeting two organelle-surface proteins involved in Ca2+ transfer at the MAM interface as previously described (10). We probed the voltage-dependent anion channel VDAC-1 at the OMM and IP3R2 at ER membrane. Each dot corresponds to one VDAC-1-IP3R2 interaction closer than 40nm, thus allowing quantification between individual islets as shown in Fig 3A and 3B. The results summarized in Table 2 and Fig 3C indicated that islets of 11 donors with Type 2 diabetes had a significant reduction in the number of dots per islet cell (mean±SEM 1.18±0.03 dots/ nucleus, n = 344 islets) in comparison to the islets of 8 controls (2.10±0.06 dots/ nucleus, n = 300 islets; Mann Whitney test, p<0.0001) with individual values shown in Fig 3D. In an independent experiment (Fig 4), we performed *in situ* PLA with islets simultaneously stained for insulin and glucagon as shown in Fig 4A and 4B. The number of dots per beta cell in donors with type 2 diabetes (nPOD # 6108, 6110, 6059, 6255) was significantly lower in comparison to controls (nPOD# 6009, 6229, 6013, 6290) with 1.47±0.07 vs 2.97±0.15 dots/ nucleus (p<0.0001), as well as the number of dots per alpha cell (0.92±0.11 vs 1.63±0.15 dots/ nucleus, p<0.001) as shown in Fig 4C. This suggests that type 2 diabetes is associated with a reduction in organelle interactions in both endocrine cell types, which was more pronounced in beta cells. There was no correlation between circulating C-peptide levels and number of dots per islet cell, as shown in S8 Fig for donors with diabetes and controls (Pearson r 0.162, p = 0.51, n = 19).

### Palmitate alters insulin secretion in Min6-B1 cells and ER-mitochondria interactions

**Fig 5:**
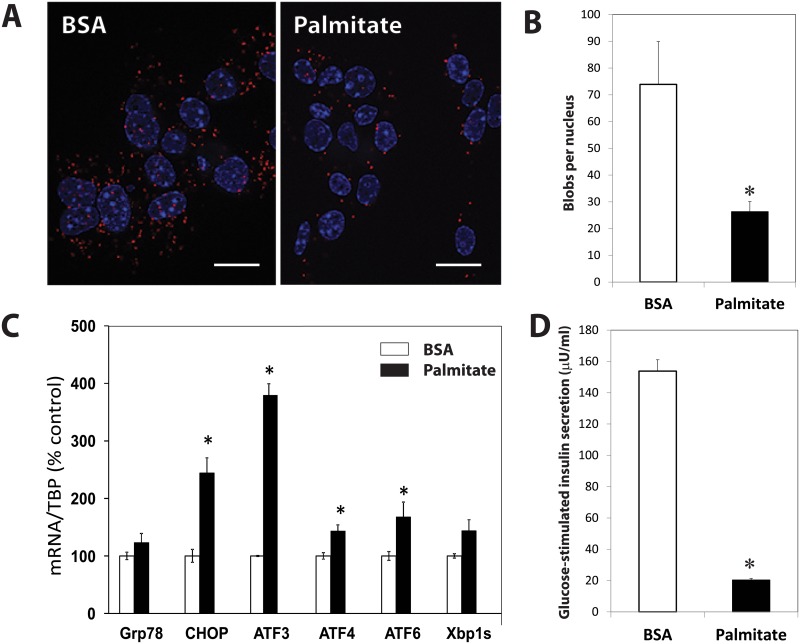
Effects of palmitate on MIN6-B1 cells. **(A)** Representative images of an *in situ* proximity ligation assay targeting IP3R1-VDAC-1 interactions of Min6-B1 cells cultured with BSA or 200 μM of palmitate for 24hrs. The scale bar indicates 25 microns. (B) Palmitate-treated MIN6-B1 cells had significatively lower ER-mitochondrial interactions. (C) Exposure of Min6-B1 cells to 200 μM palmitate (closed bars) increased the UPR response in comparison to cells cultured with BSA (open bars) and (D) reduced the glucose-stimulated insulin secretion. The asterisk corresponds to p<0.05 (student t-test).

Exposure of Min6-B1 cells to 200 μM palmitate did not modify cell viability assessed by trypan blue exclusion (90.18±0.68 vs 90.66±0.65%). Using an *in situ* PLA examining the IP3R1-VDAC-1 interactions on these cultured cells (Fig 5A, 5B and 5C), we found a significant reduction in the number of blobs per nucleus in palmitate treated cells (26.3±3.8 vs 73.9±16, p<0.04). During the same culture conditions, we compared the mtDNA/nDNA ratios. The Coxa1/ PPIA ratio was similar between palmitate-treated and BSA-treated cells (0.74±0.04 vs 0.70±0.04, p = 0.59).

We investigated during the same culture conditions, the effects of palmitate on UPR markers. As shown in Fig 5C, 24hrs incubation of MIN6-B1 cells with 200 μM palmitate increased but not significantly mRNA levels of glucose-regulated protein 78 (Grp78) (+20%) and the spliced form of X-box binding protein 1 (Xbp1s) (+44%), and significantly increased pro-apoptotic pathways through the activating transcription factor 3–4- and 6 (ATF3-ATF4-ATF6) (respectively: +279%, +44%, +67%) and the C/EBP-homologous protein (CHOP) (+144%). Insulin concentrations of both supernatants (23.71+1.4 vs 80.3+11 μU/ml, p<0.001) and acid-ethanol extracts (549.12+44 vs 1408+50 μU/ml, p<0.0001) from palmitate-treated cells were significantly lower than BSA-treated cells at 2.7mM/l glucose. In addition, palmitate treated cells had a significant reduction in GSIS (20.35±1.03 vs 153.84±7.18 μU/ml, p<0.001) (Fig 5D) and a reduction in the mean percentage of secreted insulin over insulin content from acid/ethanol cell extracts (11.0±0.88 vs 3.74±0.22, p<0.0001).

## Discussion

This is the first *in vivo* study showing differences in interactions between beta cell organelles in pancreatic islets of patients with type 2 diabetes as compared to healthy organ donors. Our data showed reduced organelle interactions despite increased expression of tethering proteins. This current study has some limitations. The most obvious is the small number of patients examined and an analysis that did not concern the whole organ. We also did not provide quantitative data on mitochondrial size and numbers. However, despite these limitations, we found significant and reproductive differences associated with type 2 diabetes with defects in organelle interplay within beta cells.

The respective contribution of reduced beta cell function vs beta cell mass in type 2 diabetes has been extensively studied. Insulin secretion in islets taken from patients with type 2 diabetes is reported to be reduced by 50% after normalization for islet insulin content [12]. In addition, diabetic islets failed to reverse hyperglycemia when transplanted to immune-deficient diabetic mice in contrast to an equivalent number of normal islets [13]. As expected, plasmatic C-peptide values were significantly reduced among the group of nPOD donors with type 2 diabetes. Different interventions such as bariatric surgery [14] or very low calorie diets [15] can lead to marked and rapid improvements of beta cell function in patients of recent onset. This confirms previous studies in first-degree relatives [16] and suggests that loss of function precedes beta cell loss and that dysfunction of beta cells is an early event of the disease.

ER is constituted by an extensive membrane network and is the principal organelle responsible for the proper folding/processing of nascent proteins in a Ca^2+^ rich environment. The architecture of the ER changes continuously according to cellular demand [17] with fusion reactions and interactions with the cytoskeleton and other organelles [18]. Here, we have demonstrated that IP3R2 expression was increased in beta cells from patients with type 2 diabetes as shown in Fig 2. IP3R2 is one of the major glucose-dependent isoforms expressed in beta cells [19], that has also the highest affinity for IP3 [20]. Increased IP3R2 amounts may reflect important changes in Ca^2+^ homeostasis and release of Ca^2+^ from the ER. We have previously shown that exposure of human islets to high glucose concentrations led to increased levels of IP3R2 both at the mRNA and protein levels, as well as reduced levels of sarco-endoplasmic reticulum Ca^2+^ ATPase 2b (SERCA 2b) pump [21]. Depletion of reticular Ca^2+^ stores results in dysfunction of protein folding activation of the unfolded protein response (UPR), which leads to ER stress and may also alter mitochondria [22]. ER Ca^2+^ release and increase in Ca^2+^ mitochondrial concentrations determine cytochrome C leaking to the cytosol, which then acts as a positive feedback loop to maintain ER Ca^2+^ release through the IP3Rs and triggers cell apoptosis [23]. Ca^2+^ also plays a key role in the regulation of mitochondrial oxidative metabolism. Mitochondrial dysfunction in beta cells can be observed after chronic exposure to high glucose concentration leading to excessive oxygen species production and reduction of mitochondrial respiration that directly alters beta cell integrity [24]. In the present study, investigation of three OMM proteins i.e. TOM20, VDAC-1 and MFN-2 gave contrasting results. This may reflect different levels of mitochondrial function and dynamics [25–27]. An increase of mitochondrial density volume may be present as already shown by EM studies [28] with enhanced mitochondrial fission and fragmentation [29]. The demonstration that expression levels of several proteins from the OMM were maintained or even increased in some patients excludes the presence of severe mitochondria alterations in beta cells of diabetic cases herein.

Since MAMs play a special role in ER-mitochondria cross talk, we investigated the numbers of tight IP3R2-VDAC-1 coupling as a marker of MAM integrity in beta cells. VDAC, a mitochondrial porin, is a highly conserved large conductance anion channel that provides the major pathway for transmembrane fluxes of ions and metabolites across the OMM [30] and is associated with cytochrome c release and apoptosis. The PLA confers dual-binder specificity for the detection of organelle interactions *in situ* and reveals proximity of proteins. We have shown previously that this technique was suitable to visualize ER-mitochondria coupling in the liver [10]. The reduction in the number of PLA dots in diabetic patients for pair of proteins involved in Ca^2+^ exchange despite an increase of IP3R2 expression shown in Fig 3 is intriguing. This may reflect the down-regulation of RE-mitochondrial interactions and tight ER-mitochondria contacts in beta cells from individuals with diabetes in comparison to controls in response to increased insulin demand. It has been shown that the subcellular distribution of the IP3R can change according to the physiological status of the cells and is an important factor for the correct initiation and propagation of Ca2+ signals [31]. Loss of MAM integrity and interactions between ER and mitochondria could be directly involved in or associated with defective insulin secretory capacities. *In vitro*, chronic fatty acid treatment of beta cells is sufficient to recapitulate both the secretory defects and apoptosis observed in type 2 diabetes. The pro-apoptotic fatty acid palmitate triggers a comprehensive ER stress response in MIN6-B1 cells [32]. Herein, it is shown that palmitate-treated Min6-B1 cells have reduced GSIS and IP3R-VDAC interactions, supporting the possible relationships between ER-mitochondrial interactions and insulin secretion. The demonstration that mtDNA/nDNA ratios were not altered by palmitate suggests mitochondrial integrity. It is still difficult to conclude that MAM dysregulation is the cause of the functional defects of beta cells during type 2 diabetes and that ER stress could be the trigger. Whether a pharmacological improvement in beta cell function involves the reinforcement of MAM integrity is at this stage too speculative, but could be evaluated *in vitro* or in animal models of diabetes.

In summary, we have demonstrated that beta cells from individuals with type 2 diabetes have reduced ER-mitochondrial interactions. We believe that this work performed with pancreatic tissue from patients is novel and represents an important advancement in the understanding of beta cell impairment with new avenues of investigation.

## Supporting information

S1 Fig IP3R2 colocalizes with beta cells. Representative immunofluorescence confocal analysis of a pancreatic section from a 55-year-old diabetic donor (nPOD# 6255) stained for insulin (green), glucagon (white) and IP3R2 (red). IP3R2 (A) is predominantly expressed in beta cells identified by insulin staining (B)and less in alpha cells stained for glucagon (GCG) (C). Merge image is shown in panel D. The scale bar indicates 50 microns.
(EPS)

S2 Fig Representative IP3R2 immunofluorescence staining. Expression patterns of IP3R2 and insulin of pancreatic islets from 12 donors with type 2 diabetes and 9 controls. The scale bar indicates 50 microns.
(EPS)

S3 Fig ITPR2 mRNA expression is increased in diabetic islets. *In situ* hybridization was performed in nPOD sample # 6255 with ITPR2 probe (A) a positive probe PPIB (B) and negative probe DapB (C). The scale bar indicates 50 microns. Morphometric analysis of ITPR2 mRNA expression in islet cells were performed in 6 donors with type 2 diabetes (closed circles) and 6 donors without history of diabetes (open circles) as shown in panel D. Each circle corresponds to the number of spots/ nucleus for a single islet. The horizontal line represents the upper 90^th^ percentile of control values.
(EPS)

S4 Fig TOM20 expression in beta cells. Representative immunofluorescence analysis in panel A with a slide scanner of pancreatic islets from a 55-year-old diabetic donor (nPOD# 6255) and a 58-year-old non diabetic donor (nPOD# 6290) for insulin (A), TOM20 (B) and merge (C). The scale bar indicates 50 microns. Panel B shows the morphometric immunofluorescence analyses of TOM20 expression in beta cells in 11 donors with type 2 diabetes (closed circles) compared to 9 donors without history of diabetes (open circles). Each circle corresponds to the mean expression level for a single islet. The horizontal line represents the upper 90^th^ percentile of control values.
(EPS)

S5 Fig Morphometric analyses of VDAC-1 expression in beta cells. Immunofluorescence studies for VDAC-1 expression in beta cells were performed with a slide scanner in 11 donors with type 2 diabetes (closed circles) compared to 8 donors without history of diabetes (open circles). Each circle corresponds to the mean expression level for a single islet. The horizontal line represents the upper 90^th^ percentile of control values.
(EPS)

S6 Fig Mitofusin-2 and VDAC-1 expression in beta cells. Representative immunofluorescence analysis of a pancreatic section with a slide scanner from a 46-year-old donor with type 2 diabetes (nPOD# 6133) and a 30-year-old non-diabetic donor (nPOD# 6235) for insulin,MFN-2,VDAC-1 and merge The scale bar corresponds to 50 microns.
(EPS)

S7 Fig Morphometric analysis of mitofusin 2 expression in beta cells. Immunofluorescence studies for MFN-2 expression in beta cells were performed with a slide scanner in 11 donors with type 2 diabetes (closed circles) compared to 9 donors without history of diabetes (open circles). Each circle corresponds to the mean expression level for a single islet. The horizontal line represents the upper 90^th^ percentile of control values.
(EPS)

S8 Fig Absence of correlation between the number of IP3R2- VDAC1 complexes and peripheral C-peptide levels. Mean results of *in situ* proximity ligation assay per islet cells do not correlate with peripheral C-peptide levels both in donor with diabetes (closed symbols) or controls (open symbols).
(EPS)
